# Genome-Editing Products Line up for the Market: Will Europe Harvest the Benefits from Science and Innovation?

**DOI:** 10.3390/genes15081014

**Published:** 2024-08-01

**Authors:** Alexios Polidoros, Irini Nianiou-Obeidat, Nikolaos Tsakirpaloglou, Nestor Petrou, Eleftheria Deligiannidou, Nefeli-Maria Makri

**Affiliations:** Laboratory of Genetics and Plant Breeding, Faculty of Agriculture, Aristotle University of Thessaloniki, 54124 Thessaloniki, Greece; nianiou@agro.auth.gr (I.N.-O.); n.tsakirpaloglou@gmail.com (N.T.); nestorpetrou@gmail.com (N.P.); rikadel22@gmail.com (E.D.); nefelimmmakri@gmail.com (N.-M.M.)

**Keywords:** CRISPR, crop improvement, European Union (EU) regulation, Genetically Modified Organisms (GMOs), New Genomic Techniques (NGTs), product commercialization

## Abstract

Clustered Regularly Interspaced Short Palindromic Repeats (CRISPR) technologies have revolutionized genome editing, significantly advancing the improvement of cultivated crop species. This review provides an overview of genome-edited crops that have either reached the market or received the necessary approvals but are not yet available to consumers. We analyze various genome-editing studies to understand the distribution of different genome-editing systems, the types of site-directed nucleases employed, and the geographical spread of these studies, with a specific focus on global and European contexts. Additionally, we examine the target crops involved. The review also outlines the multiple steps required for the legal acceptance of genome-edited crops within European jurisdictions. We conclude with suggestions for the future prospects of genome-editing research in Europe, aiming to streamline the approval process and enhance the development and adoption of genome-edited crops.

## 1. Introduction

**Figure 1:**
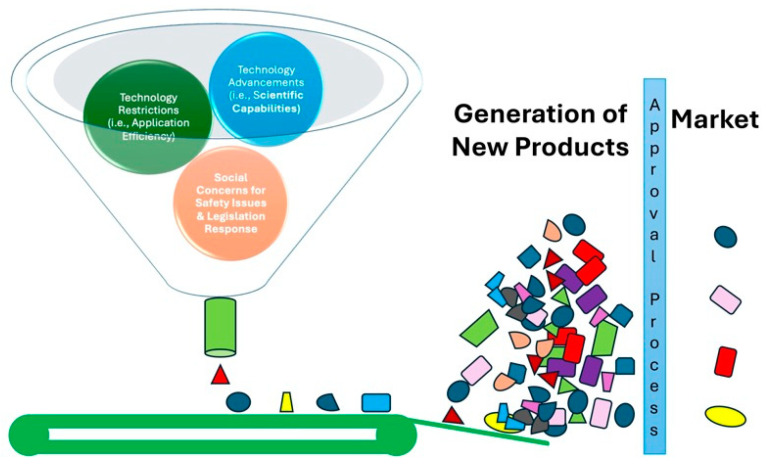
Genome Editing: Tiers from scientific discoveries to product development. The conceptual framework for the translation of scientific advancements of genome editing into tangible products is presented. The funnel represents the narrowing range of possibilities as discoveries progress through different stages. As these advancements narrow down the funnel, they are filtered through a series of restrictions, including technological limitations, application efficiency, and social concerns around safety. Finally, a select few advancements reach the bottom of the funnel, where they undergo a market approval process before reaching the market as new products.

Clustered Regularly Interspaced Short Palindromic Repeats (CRISPR) technologies have fundamentally transformed genome editing (GE), offering applications that extend well beyond consumer-oriented advantages. While CRISPR-edited foods have captured considerable attention, the potential impact of CRISPR-edited crops transcends the realm of the supermarket [1]. These crops, modified using CRISPR without the introduction of foreign DNA, possess numerous benefits. They bolster resilience to climate change, aiding in the adaptation of current crop varieties and ensuring agricultural productivity remains robust under adverse conditions. Additionally, localized crop varieties stand to benefit from targeted CRISPR modifications, which enhance disease resistance, nutrient profiles, and yield, thereby fortifying farmer livelihoods and food security. Furthermore, CRISPR-edited crops engineered for pest and disease resistance can curtail the use of chemical pesticides, offering dual benefits for human health and the environment. Notably, the regulatory landscape for these cis-edited crops differs from that of genetically modified organisms (GMOs), as they do not involve foreign gene insertion, simplifying their adoption. Importantly, CRISPR’s precision preserves crop genetic diversity, vital for resilience against environmental shifts and evolving pests. In summary, CRISPR-edited crops present a promising frontier for sustainable agriculture, global food security, and climate resilience, highlighting their potential to significantly benefit both producers and consumers alike (Figure 1).

### Genome Editing and Engineered Nucleases

Genome editing, also known as gene editing or genome engineering, encompasses a collection of powerful techniques called New Genomic Techniques (NGTs) for the precise modification of the DNA in living organisms. This revolutionary technology empowers scientists to insert, delete, modify, or replace specific sequences within an organism’s genome, offering unprecedented control over the genetic code. The core mechanism of genome editing hinges on the utilization of programmable nucleases, enzymes engineered to recognize and bind to designated genomic targets. These nucleases, equipped with dedicated DNA-binding domains, strategically target specific sequences within the genome. Upon binding, they introduce precise double-strand breaks (DSBs) at the designated location. Subsequently, the cell’s inherent DNA repair machinery takes over, attempting to mend the broken strands. Two primary repair pathways can be exploited for genome editing: Homology-Directed Repair (HDR) and Non-Homologous End Joining (NHEJ). HDR utilizes a similar DNA sequence as a template to guide the repair process, enabling the precise insertion of new genetic information or correction of existing mutations. NHEJ, on the other hand, results in the deletion or insertion of small DNA segments, which can still be harnessed for specific editing purposes. By manipulating these repair pathways, it is possible to achieve diverse genetic modifications, paving the way for advancements in gene therapy, disease modeling, and various other research applications [2,3].

The development of engineered nucleases—enzymes capable of cleaving DNA at specific target sequences—has revolutionized genome editing. These nucleases initiate precise DSBs within the genome, stimulating cellular DNA repair mechanisms that can introduce targeted modifications [4]. Currently, four major classes of engineered nucleases exist: zinc-finger nucleases (ZFNs), transcription activator-like effector nucleases (TALENs), meganucleases (engineered homing endonucleases), and the CRISPR/Cas9 system.

ZFNs were the first programmable enzymes designed to introduce precise modifications to the genomes of plants and animals, by recognizing and cleaving DNA at specific locations [5,6]. They consist of two key domains: a DNA-binding domain composed of zinc finger (ZF) modules and a cleavage domain derived from the FokI enzyme [6]. Each ZF module recognizes a specific 3-base sequence in DNA, allowing for the modular assembly of ZFNs to target longer sequences, typically 18–24 nucleotides sequence. Notably, the FokI cleavage domain requires the dimerization of ZFNs bound to opposite strands of the target DNA ensuring precise double-strand breaks only at the intended location [7]. This targeted cleavage facilitates various genome-editing strategies, highlighting the versatility of ZFNs as powerful tools in genetic engineering.

Similar to ZFNs, TALENs are versatile tools for precise genome editing in different organisms [8,9]. TALENs utilize a distinct DNA-binding domain derived from the transcriptional activator-like effector (TALE) proteins produced by plant-pathogenic *Xanthomonas* bacteria [8]. These TALEs possess an array of repetitive amino acid sequences (typically 13–18 copies of a 34-amino acid repeat), where each repeat specifically recognizes one nucleotide in the DNA code [10,11]. Remarkably, by manipulating the composition of these repeats, TALENs can be engineered to bind to virtually any desired DNA sequence, offering unparalleled targeting flexibility compared to ZFNs. Like ZFNs, TALENs employ the FokI nuclease for DNA cleavage, ensuring targeted double-strand breaks at the designated genomic location [8,9]. This combination of customizable DNA binding and precise cleavage empowers TALENs as powerful instruments for various genome-editing applications.

Meganucleases, unlike their engineered counterparts (ZFNs and TALENs), are naturally occurring enzymes possessing both DNA recognition and cleavage capabilities [12,13]. These enzymes originate from mobile genetic elements known as homing endonucleases (HEs) found within introns [13]. Unlike the modular design of ZFNs and TALENs, meganucleases recognize and cleave DNA at specific locations typically as dimers of two identical subunits or through a single peptide domain [14]. This domain exhibits remarkable specificity, targeting extended DNA sequences ranging from 12 to 40 nucleotides, a significantly longer recognition sequence compared to ZFNs and TALENs [12,13]. The inherent rarity of these long target sequences within the genome contributes to the exceptional specificity of meganucleases, making them valuable tools for precise genome-editing applications.

The CRISPR/Cas9 system has emerged as a transformative technology in genome editing [15,16]. Originally a bacterial defense mechanism against invading viruses and plasmids, CRISPR/Cas9 leverages its inherent DNA targeting and cleavage capabilities for diverse applications in research, medicine, and agriculture [17,18]. The system relies on a dual RNA molecule complex, naturally consisting of CRISPR RNA (crRNA) and trans-activating CRISPR RNA (tracrRNA) [19]. Recognition of specific DNA sequences hinges on the presence of a short protospacer adjacent motif (PAM) and complementary base pairing between a single-guide RNA (sgRNA)—a synthetic mimic of the crRNA-tracrRNA complex—and the target DNA. This intricate interplay between Cas9, RNA elements, and target DNA facilitates precise double-stranded breaks, enabling targeted genome modifications, transcriptional control, epigenetic alterations, and even visualization of specific genomic loci [3,20]. The remarkable simplicity and programmability of CRISPR/Cas9 have revolutionized diverse fields, solidifying its position as a powerful and versatile tool for scientific exploration and potential crop improvement interventions.

Moreover, advancements in genome editing have opened new avenues for crop improvement. The development of novel tools like prime editing [21,22,23], site-directed nucleases (including CRISPR–Cas systems) [24], and oligonucleotide-directed mutagenesis [25], and bridge RNA-guided recombination [26] has significantly enhanced our ability to precisely modify crop genomes. Additionally, the ongoing discovery of new enzymes [26,27,28] with unique editing capabilities in other organisms holds immense promise for further revolutionizing crop breeding.

## 2. Crop Improvement through Genome Editing

### 2.1. Approved Crops—Genome Editing

The advent of powerful gene-editing technologies, particularly CRISPR–Cas9 and TALENs, has fundamentally transformed the field of crop improvement. These tools facilitate precise, targeted modifications to plant genomes, offering unprecedented control over diverse traits critical for addressing global agricultural challenges, such as food security and nutritional deficiencies, and adapting to the pressures of climate change. While numerous gene-edited crops have gained regulatory approval worldwide, only a select few have achieved commercialization thus far (Table 1). Initial commercialized traits prioritize improvements in shelf life, enhancing nutritional value, and modifying oil composition to meet consumer and industry demands.

A significant emphasis in commercialized gene-edited crops lies in extending shelf life and reducing food waste. This focus builds upon earlier efforts utilizing RNAi technology to disrupt browning reactions in apples and potatoes [29,30], which led to the first wave of genetically modified (GM) products addressing this issue. However, genome editing has revolutionized the approach, enabling the development of a wider range of non-browning fruits and vegetables, including bananas, lettuce, and mustard greens [31,32,33,34,35,36,37,38]. Unlike traditional GM techniques, genome editing achieves extended shelf life through precise modifications to existing plant genes rather than introducing foreign DNA. This distinction offers economic benefits for producers and retailers, along with improved accessibility and a potentially greater sense of familiarity for consumers seeking fresh produce.

Beyond improving aesthetics, gene editing offers a powerful tool for developing crops with nutritionally optimized profiles. A prime example is the creation of high γ-aminobutyric acid (GABA) tomatoes [39,40,41,42,43,44,45,46,47]. These engineered tomatoes boast significantly elevated levels of this neuroactive amino acid, which has been linked to relaxation and potentially lower blood pressure. The health advances become even more attractive when these tomatoes are combined with increased anthocyanin content, a type of antioxidant associated with numerous health benefits [44,45,46,47]. Furthermore, this technology has enabled the creation of high oleic acid soybean oil, showcasing its versatility in modifying fatty acid profiles to generate healthier and more stable cooking oils [48,49,50,51]. These advancements highlight the diverse and far-reaching potential of gene editing in fostering a more nutritious and sustainable food system.

Gene editing demonstrates remarkable versatility, proving its value not only in enhancing consumer food products but also in addressing the specialized needs of various industries. The development of canola and soybean oils with precisely tailored fatty acid profiles exemplifies the power of gene editing to optimize oil composition for demanding applications [51,52,53]. Through genome editing, vegetable oils can be engineered to be high-oleic (HO). These HO oils offer a healthier alternative to currently used fats and oils containing trans fatty acids (TFAs) [54]. TFAs have been linked to negative health outcomes, and widespread adoption of HO oils could significantly improve population-level fatty acid profiles. Furthermore, precise editing of the waxy gene has yielded a corn variety with nearly 100% amylopectin starch content [55,56,57,58]. This waxy corn starch, with its unique properties, finds valuable applications in the papermaking and adhesive industries and as a natural stabilizer and thickener in the food industry. This strategic use of gene editing unlocks an exciting new avenue in the creation of sustainable, plant-based resources. It has the potential to revolutionize how we source materials, offering replacements for less environmentally friendly alternatives across a wide range of industries.

Gene-edited crops have already made their way to the market, but many more innovations are in the pipeline, awaiting commercial approval [59]. Some of the most remarkable examples include crops exhibiting enhanced resistance to diseases and biotic stresses, as well as improved postharvest properties (Table 1). For instance, genome editing has enabled the creation of wheat varieties that resist the fungal pathogen powdery mildew (*Blumeria graminis* f. sp. *tritici* (Bgt)) [60], which is one of the most destructive plant pathogens worldwide, thereby reducing crop losses and pesticide use. Similarly, targeted disruptions of soybean genes involved in abiotic stress responses have resulted in increasing its tolerance to drought and salinity, making it more adaptable to climate change [61]. Furthermore, gene editing has also been applied to modify post-harvest traits, such as the ripening, color, and firmness of fruits, in a variety of crops, including white button mushrooms, potatoes, wheat, alfalfa, and false flax, resulting in better quality and less food waste [61,62,63,64,65].

**Table 1:** Presentation of genome-edited products/crops that have been commercialized or received approval but not yet commercialized, globally. This list is based on the information presented at the Global Gene Editing Regulation Tracker of the Genetic Literacy Project (https://crispr-gene-editing-regs-tracker.geneticliteracyproject.org/united-states-crops-food/) (accessed on 25 July 2024) and the first decade of CRISPR: Advances and Outlook (https://www.isaaa.org/blog/entry/default.asp?BlogDate=2/21/2024) (accessed on 25 July 2024). In the columns “Link” and “Patent Lens”, there are clickable links as “ref” and “Link”, respectively, to websites with the relevant information (last checked on 4 July 2024).

	Year	Crop	Trait	GE Technique	Organization	Notes	Link	Patent Lens
A. Commercialized Products								
1.	2023	Banana (*Musa* spp.)	Reduced browning	CRSPR-Cas9	Tropic Biosciences UK, Ltd.	The reduced browning GE banana was determined to be a non-GMO by the Philippines Department of Agriculture-Bureau of Plant Industry. This banana is the first gene-edited product to go through the Philippines’ gene-editing regulatory process.	[31,33]	https://www.lens.org/lens/patent/089-116-471-909-450/fulltext?l=en (accessed on 25 July 2024)
2.	2023	Lettuce (*Lactuca sativa*)	Non-browning lettuce	CRISPR–Cas9	GreenVenus, Llc.	The romaine lettuce has improved shelf life up to two weeks. The lettuce plants have combinations of polyphenol oxidase (“PPO”) gene mutations to reduce browning, reduce tip burn, create longer shelf life, and improve nutrition as compared to non-mutated varieties.	[35]	https://www.lens.org/lens/patent/185-656-263-620-844/fulltext?l=en (accessed on 25 July 2024)
3.	2023	Corn (*Zea mays*)	Amylopectin-enriched waxy corn	CRISPR–Cas9	Pioneer Hi Bred Int	Japan approved a high-starch corn variety, the fourth GE food product that Japan did not subject to regulations for GMO crops. The waxy gene in the said corn variety was deleted using CRISPR–Cas9 technology to increase its starch amylopectin proportion to almost 100%. Approval for commercialization in the USA is pending.	[33,55,56]	https://www.lens.org/lens/patent/066-043-105-670-954/frontpage?l=en (accessed on 25 July 2024) https://www.lens.org/lens/patent/099-921-177-981-376/frontpage?l=en (accessed on 25 July 2024)
4.	2023	Brassica (*Brassica oleracea*)	Less pungent mustard greens	CRISPR–Cas9	Pairwise Plants Services, Inc.	Produced by knocking out of all functional copies of the type-I myrosinase multigene. It is marketed as “Conscious Greens”, that have the taste and texture of lettuce, but double the nutrition of Romaine and upwords of three extra days of shelf. Discontinued in the USA, shortly after its debute.	[37,38]	https://www.lens.org/lens/patent/143-473-546-918-944/frontpage (accessed on 25 July 2024)
5.	2021	Tomato (*Lycopersicon esculentum*)	Purple tomato with high γ-aminobutyric acid (GABA)	CRISPR–Cas9	Norfolk Healthy Produce, Ltd.	The tomato host intended increased levels of anthocyanins (increased antioxidant properties), which also results in its harmless purple color. Norfolk Healthy Produce is a subsidiary of the John Innes Center.	[44,45,46]	https://www.lens.org/lens/patent/172-128-558-724-105/frontpage?l=en (accessed on 25 July 2024)
6.	2021	Tomato	High levels of GABA	CRISPR–Cas9	Sanatech Seed Co, Ltd.	The variety “Sicilian Rouge High GABA” contains high levels of γ-AminoButyric Acid (GABA), an amino acid believed to aid relaxation and help lower blood pressure.	[39,40,41,42,43]	
7.	2019	Soybean (*Glycine max*)	Oil with high-oleic acid, less saturated fat, and no trans-fat	TALENs	Calyxt, Inc.	Calyno™ oil was developed by knocking out the fatty acid desaturase genes FAD2-1A and FAD2-1B in soybean through genome editing. The TALENs-edited soybeans produce oil that contains 80% higher oleic acid, 20% less saturated fatty acids, has zero grams trans fat per serving, has three times the fry-life and has a longer shelf-life compared to the current soybean oil being sold in the market. (Calyxt, Inc. merged with Cibus, Inc. on 1 June 2023)	[48,49,50]	https://www.lens.org/lens/patent/097-648-435-235-791/fulltext?l=en (accessed on 25 July 2024)
8.	2015	Canola (*Brassica napus*)	Oil with high oleic acid	TALENs	Cibus, Inc.	SU Canola™ is the first non-transgenic, genome-edited crop approved in the US and commercialized on 10,000 acres (4000 hectares). The product was not required by USDA to pass through the usual GM regulation in the USA.	[52,54]	https://www.lens.org/lens/patent/034-948-031-959-311/frontpage?l=en (accessed on 25 July 2024)
B. Approved, Not Yet Commercialized								
1.	2022	Pennycress (*Thlaspi arvense*)	High yield	CRISPR–Cas9	Covercress, Inc.	This pennycress variety has been developed to be higher in oil and lower in erucic acid, a fatty acid that is not good for human health. It was created using CRISPR–Cas9 and is currently being developed by Covercress. The crop is FDA approved, but commercialization is pending.	[66]	https://www.lens.org/lens/patent/046-467-999-135-312/fulltext?l=en (accessed on 25 July 2024)
2.	2020	Corn	Higher yield waxy corn	CRISPR–Cas9	Corteva Agriscience	Using CRISPR–Cas9 gene editing researchers from Corteva Agriscience created corn hybrids with superior performance to those obtained using modern trait introgression methods.	[56]	
3.	2018	Wheat (*Triticum aestivum*)	High-fiber wheat		Calyxt, Inc.	Calyxt developed the wheat as a healthier wheat option. Cleared by the USDA, but commercialization is pending.	[65]	
4.	2017	Camelina (*Camelina sativa*; false flax)	Enhanced omega-3 oil content	CRISPR–Cas9	Yield10 Bioscience, Inc.	Developed using CRISPR and cleared by the USDA. Camelina with increased oil content; target genes not disclosed.	[61]	https://www.lens.org/lens/patent/101-585-593-170-871/frontpage?l=en (accessed on 25 July 2024)
5.	2017	Soybean	Drought- and salt-tolerant	CRISPR–Cas9	USDA ARS, Plant Science Research Unit	Soybean (Glycine max) with drought and salt tolerance; achieved by disrupting the Drb2a and Drb2b genes (double-stranded RNA-binding protein2 genes).	[61]	
6.	2017	Alfalfa (*Medicago sativa*)	Improved quality	TALENs	Calyxt, Inc.	Developed by Calyxt using TALENs; designated by the USDA as non-regulated.	[64]	
7.	2017	Wheat	Powdery mildew-resistant	TALENs	Calyxt, Inc.	Developed by Calyxt using TALENs; designated by the USDA as non-regulated in 2016. Field trials began in 2017.	[60]	
8.	2017	Setaria (*Setaria viridis*)	Delayed flowering time	Unknown	Donald Danforth Plant Science Center	Setaria viridis, or green bristlegrass, with delayed flowering time; achieved by deactivating the S. viridis homolog of the Zea mays ID1 gene.	[61]	
9.	2016	Potato (*Solanum tuberosum*)	Non-browning	TALENs	Calyxt, Inc.	Developed by Calyxt using TALENS and cleared by the USDA in 2016.	[63]	
10.	2016	Mushroom (*Agaricus bisporus*)	Resistant to browning	CRISPR–Cas9	Penn State University	Developed at Pennsylvania State University using CRISPR and designated by the USDA as non-regulated. White button mushroom (Agaricus bisporus) with antibrowning properties; achieved by knocking out a gene coding for polyphenol oxidase (PPO).	[62]	
11.	2016	Corn	Corn with extra starch (waxy corn)	CRISPR–Cas9	DuPont	Corn with high starch content (waxy maize) developed by DuPont using CRISPR planted in test fields. Designated by the USDA as non-regulated, but not introduced commercially. Waxy corn with starch composed exclusively of amylopectin; achieved by inactivating the endogenous waxy gene Wx1 that encodes a granule-bound start synthase catalyzing production of amylose.	[61]	

### 2.2. Technological and Geographical Distribution of Genome Editing Technology

**Figure 2:**
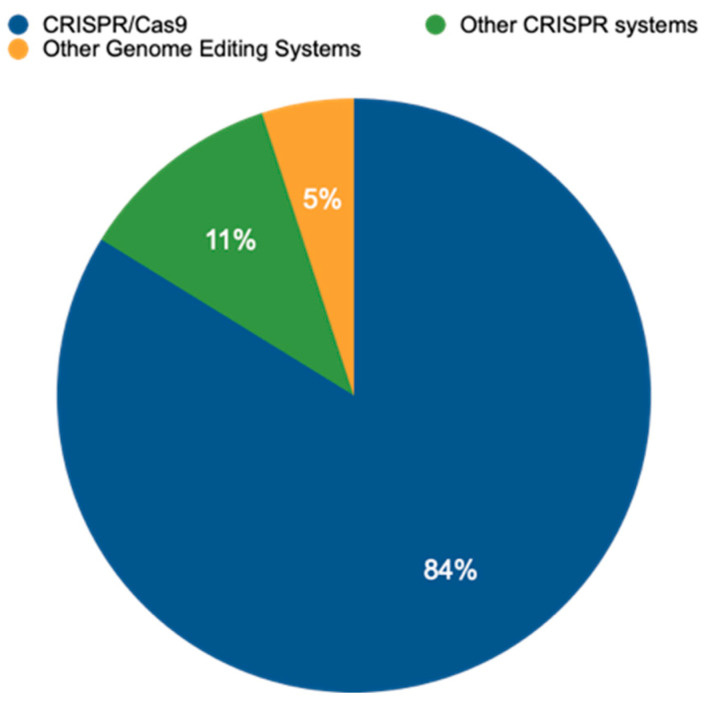
Distribution or relative prevalence of different genome-editing techniques in a set of 837 studies. CRISPR/Cas9 is currently the most prevalent method, allowing researchers to make precise changes to an organism’s DNA. Other CRISPR systems, including Cas12, Cas12a, and Cas13, offer variations on this approach and offer researchers additional functionalities. Beyond CRISPR, several other genome-editing systems have been developed, including oligonucleotide-directed mutagenesis (ODM), Zinc Finger Nucleases (ZFNs), Transcription Activator-Like Effector Nucleases (TALENs), Site-Directed Nuclease 3 (SDN-3), prime editing (PE), and base editing (BE), offering even greater precision and enabling researchers to insert specific DNA sequences or directly convert single nucleotides within the genome without creating double-strand breaks.

An analysis of publicly available data from the EU-SAGE database (https://www.eu-sage.eu/genome-search) (accessed on 25 July 2024)—a collection of information on genome-edited crop plants documented in scientific studies—revealed that CRISPR–Cas systems have emerged as the dominant method for genome editing. This powerful technology allows researchers to modify an organism’s DNA with high precision. Our analysis of 837 studies (Table S1) revealed that CRISPR/Cas systems accounted for a staggering 95% of all genome-editing techniques employed within the analyzed data (Figure 2). This dominance is further highlighted by the prevalence of CRISPR/Cas9, the most widely used variant within the CRISPR/Cas family, representing 83.8% of all CRISPR/Cas applications. While CRISPR/Cas9 reigns supreme, this study also identified the use of other CRISPR variants, including CRISPR/Cas12 (1.6%), base editing (2.2%), CRISPR/Cas13 (0.5%), and CRISPR/Cas12a (0.5%). Notably, the data also revealed the presence of alternative genome-editing techniques, such as TALENs at 3.2% and ZFNs at 0.7%. These findings underscore the remarkable versatility of CRISPR-based methods while acknowledging the continued presence of alternative approaches in the field of genome editing.

In addition, analysis of the data reveals a clear preference for site-directed nucleases 1 (SDN-1), which accounts for a remarkable 93.9% of all SDN usage. This dominance suggests that SDN1 offers significant advantages for researchers seeking to modify genes in living organisms. While not as prevalent, Base Editing (BE) demonstrates a growing presence at 2.2%. This technique allows for targeted modifications of single nucleotides within DNA, showcasing its potential for precise editing applications. SDN-2 and SDN-3, on the other hand, are employed to a lesser extent at 1.2% and 1.0%, respectively. These techniques likely address specific editing needs that SDN1 or BE may not fully satisfy. Finally, Oligonucleotide-Directed Mutagenesis (ODM) represents the least utilized technique, at only 0.8%. This finding suggests that ODM may be more specialized compared to the other SDN methods. In conclusion, these data highlight the overwhelming dominance of SDN1 in genome editing. Its widespread adoption underscores its effectiveness and ease of use for researchers. However, the presence of alternative techniques like BE demonstrates the ongoing development and diversification within the field of SDN-based genome editing.

**Figure 3:**
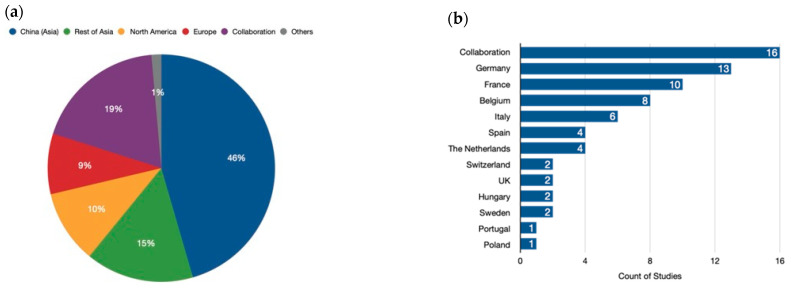
Geographical distribution of genome-editing research. (**a**) Global distribution. China (Asia) leads in the number of publications related to genome-editing research. However, international collaboration is also a significant driver of progress, with many publications resulting from joint efforts by researchers from different continents. The category labeled “Others” represents the contributions of Africa and Oceania to this field. (**b**) European distribution. While global collaboration is a key player, Europe also boasts a strong network of research focused on genome editing. It is evident that collaborations among European countries are the main driving force for the published research studies in plant-genome-editing projects. Additionally, Germany and France stand out as individual contributors with a significant number of publications.

The analysis revealed a significant concentration of research efforts in Asia, particularly China, which accounted for nearly half (45.3%) of all studies (Figure 3a). Our analysis also highlights the growing importance of international collaboration in genome-editing research. Almost one-fifth (18.7%) of the analyzed studies involved collaboration between researchers from different continents. This collaborative approach is crucial for accelerating scientific progress and ensuring that the benefits of genome-editing technologies are shared globally. Following China, other regions in Asia collectively contributed 15.4% to the analyzed research. North America and Europe followed closely behind, with contributions of 10.4% and 8.6%, respectively. Moreover, Africa, Oceania, and Latin America collectively accounted for a smaller proportion of the research efforts, with each continent contributing less than 1% of the analyzed studies. This underscores the need for increased investment and infrastructure development in these regions to foster a more balanced global landscape of genome-editing research. In conclusion, our analysis sheds light on the geographic distribution of genome-editing research efforts. While Asia, particularly China, is currently at the forefront of this field, international collaboration is playing an increasingly important role. Continued investment and global partnerships are essential to ensure equitable access to this powerful technology and its applications.

Our analysis of genome-editing research in Europe reveals a diverse landscape, with countries exhibiting varying levels of involvement and collaboration (Figure 3b).

Germany takes the lead, contributing a substantial 18.3% of the continent’s overall efforts. This robust research infrastructure positions them as a frontrunner in European genome editing. France follows closely behind with 14.1%, demonstrating a strong national commitment to the field. Beyond these leaders, Belgium (11.3%) and Italy (8.5%) emerge as significant contributors, underscoring the presence of active research hubs across Europe. Spain and the Netherlands (each at 5.6%) also display noteworthy participation. Notably, the United Kingdom, Switzerland, Sweden, and Hungary, while still involved, exhibit a more moderate level of engagement, each contributing around 2.8%. Intriguingly, collaboration across European borders plays a vital role in advancing the field. A remarkable 21.1% of the research efforts involve collaboration between European countries. This collaborative spirit is particularly evident between Belgium and France, whose frequent partnerships contribute an additional 2.8% to the joint research pool. This emphasis on international cooperation highlights the importance of fostering a pan-European research environment to propel the development and responsible application of genome-editing technologies.

**Figure 4:**
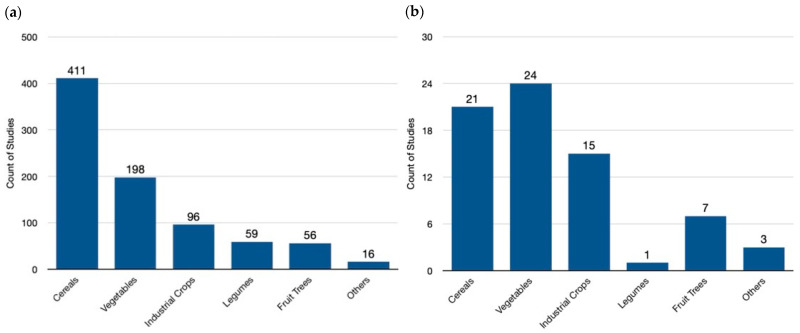
Crop species targeted in genome-editing research: a global and European perspective. (**a**) A global view in the field of genome-editing research, highlighting cereals—like wheat, rice, and maize—as the leading focus area. This trend reflects the potential of genome editing to address global food security challenges by improving staple crops. (**b**) The distribution in the field of genome-editing research within Europe. Here, a noticeable shift towards vegetables is evident, suggesting researchers might be tailoring their efforts towards crops with regional importance or specific dietary needs. Finally, the “Others” category encompasses a diverse array of specific species of plant families with no common classification, including Asteraceae (Chrysanthemum, Rubber Dandelion), Brassicaceae,(Ornamental kale, Pennycress), Convolvulaceae (Morning glory), Euphorbiaceae (Poinsettia), Lamiaceae (Sage, Sweet Basil), Linderniaceae (Torenia), Papaveraceae (Opium poppy), and Solanaceae (Petunia) families. This variety underscores the broad potential applications of genome editing, extending beyond staple crops and encompassing a wider range of agricultural interests.

Our analysis of the available genome-editing studies reveals a clear prioritization of cereal crops, which constitute nearly half (49.0%) of all endeavors globally (Figure 4a). This focus reflects the importance of staple food crops in ensuring global food security. Following closely are vegetables (23.6%), likely due to their diverse applications and potential for yield improvement. Industrial crops (11.5%) also hold a significant share, reflecting their economic importance in biofuel production and industrial materials. Legumes (7.0%) and fruits/trees (6.9%) represent smaller but noteworthy portions, potentially targeting improved nutrition or adaptation to changing environments. A minor category encompassing weeds, herbs, and ornamentals (1.9%) highlights the ongoing exploration of this technology for diverse plant applications.

In contrast, the European landscape of genome-edited crops (Figure 4b) showcases a distinct prioritization compared to the global trend. Here, the primary focus lies on vegetables (33.8%), particularly those belonging to the Solanaceae family (tomato, potato, eggplant, etc.) This emphasis might be driven by Europe’s significant vegetable production and consumption, as well as the amenability of these crops to genetic modification techniques. Cereals remain important at 30.6%, reflecting their role in European food security. However, the focus is slightly lower compared to the global level. Industrial crops (21.1%) garner substantial attention in Europe, potentially due to their role in bio-based product development and a push for sustainable industrial practices. Fruits/trees (9.9%) receive moderate emphasis, while legumes (1.4%) receive less focus compared to the global landscape. A small “others” category (4.2%) encompasses a wider range of plant types in Europe.

A comparison of global and European data reveals distinct research priorities in genome editing. Globally, cereals dominate, reflecting the critical role of staple food security. In contrast, Europe places a greater emphasis on vegetables, potentially driven by specific regional dietary preferences and established production systems. Industrial crops also exhibit a difference, receiving a higher share of research focus in Europe, which might be linked to its bioeconomy goals. Fruits/trees maintain a similar representation across both regions, suggesting a balanced approach to improving these long-lived crops. Notably, legumes receive considerably less attention in Europe compared to the global landscape. This could be due to various factors, such as existing breeding programs or a lower economic incentive for legume improvement in the European context.

## 3. The European Union Legislative Procedure to Regulate New Genomic Techniques and Their Products

It is important to note that while there are only a handful commercially available products thus far, there are many other genome-edited crops in the pipeline, with potential applications in areas such as improved nutrition, disease resistance, stress tolerance, and increased yield. The regulatory framework for genome-edited crops is continually developing, and it is increasingly apparent that there is a shared commitment among nations to distinguish between transgenics and genome-edited plants. Currently, several countries, such as the USA, Canada, Japan, Australia, Argentina, Brazil, Israel, India, and others, consider genome-edited plants that do not contain foreign DNA as conventionally bred varieties. Furthermore, several other countries like China, Russia, and the European Union (EU) are planning legislative reforms to distinctly regulate genome-edited plants separate from genetically modified organisms, aligning with the broader global movement in this direction.

### 3.1. The GMO Legislation Revision in the EU

When the necessity for a new legislation is recognized in the EU, the European Commission (EC) is responsible for submitting a legislative proposal and engages in a consultative process involving member states, the European Parliament, stakeholder organizations, expert groups, and the public to gather opinions and feedback on its legislative proposals. Subsequently, the EC submits the proposal to both the Council and the European Parliament. Following this, the Council and the Parliament proceed to adopt the legislative proposal, which can occur during either the first or second reading stages where modifications are also suggested. However, should the two institutions fail to reach an agreement after the second reading, a conciliation committee, a “trilogue”, is convened to resolve any remaining differences. A trilogue is an informal interinstitutional negotiation wherein representatives from the European Parliament, the Council of the European Union, and the European Commission come together to agree to a final text [67].

This process is currently underway to enact new legislation governing NGT-derived plants and their products (Table 2). The current GMO legislation within the EU traces back to 2001, with the issuance of Directive 2001/18 [68], which addressed the deliberate release of genetically modified organisms into the environment, replacing the initial Council Directive 90/220/EEC. Subsequent regulations addressing labeling, traceability, and GMO isolation have supplemented the EU’s GMO legislation. Over the past decade, numerous NGTs have emerged, primarily based on advancements in genome-editing technologies like TALENs and CRISPR/Cas [69]. Debates surrounding the regulatory framework for these new plant breeding techniques (NBTs, currently referred to as NGTs), have been ongoing since 2008, particularly questioning whether all their products fit within the general legal definition of GMOs. Despite repeated requests from academia and businesses, the Commission failed for a long time to provide clarity on the regulatory status of NGT-derived plant varieties [70].

The absence of a response regarding the revision of the GMO regulatory status for NGT-derived plant varieties that have genetic modifications excluding the insertion of foreign DNA experienced an unexpected turn of events in 2015. At that time, nine French NGOs and farmers’ unions initiated legal action before the highest French administrative court, questioning the legal status of two new herbicide-tolerant varieties as “hidden GMOs”, claiming that they might be NGT-developed, employing ‘directed mutagenesis’ or ‘targeted mutagenesis’ techniques [71]. In October 2016, the higher French Court sought the European Court of Justice’s (ECJ) opinion on whether mutagenized varieties should be classified as GMOs, leading to the ECJ’s July 2018 opinion confirming NGT-derived organisms as GMOs. This placed them under the EU-wide regulatory oversight set by the Directive 90/220 for GMOs (https://eur-lex.europa.eu/legal-content/EN/TXT/?uri=CELEX%3A31990L0220) (accessed on 25 July 2024). The ECJ’s decision prompted concerns about innovation stagnation and enforcement challenges, with some foreign countries adopting different regulatory approaches. Responding to those concerns and studies that highlighted the potential of NGT-developed plants to address various needs, in November 2019, the European Council requested the Commission to conduct a study on the novel genomic techniques, which was delivered on 29 April 2021. The study emphasized the potential contributions of NGT products to sustainable food systems and a competitive economy, while addressing concerns about safety, environmental impacts, and labeling.

The study suggested the need to adapt the 2001 GMO legislation to certain NGTs, leading to a proposal for updated NGT regulation, which was prepared to take into account suggestions and opinions after public consultation, the European Food and Safety Authority (EFSA)’s opinion on the risk assessment criteria for plants produced by targeted mutagenesis, cisgenesis, and intragenesis, and the mandatory impact assessment by the Regulatory Scrutiny Board.

The proposal distinguished **NGT Category 1** plants, which are “considered equivalent to conventional plants”, and **NGT Category 2**, which includes all other plants obtained through NGTs. The proposal considers an NGT plant equivalent to conventional plants according to specific criteria detailed in Box 1. NGT Category 2 plants are defined by default: “They include all other varieties obtained through NGTs”. Plants that have been modified to carry foreign DNA, regardless of the technique employed, fall under the current GMO legislation and are treated as transgenic plants.

Box 1 **Criteria of equivalence of NGT plants to conventional plants.**

An NGT plant is considered equivalent to conventional plants when it differs from the recipient/parental plant by no more than 20 genetic modifications of the types referred to in points 1 to 5, in any DNA sequence sharing sequence similarity with the targeted site that can be predicted by bioinformatic tools.

    (**1**) substitution or insertion of no more than 20 nucleotides;
    (**2**) deletion of any number of nucleotides;
    (**3**) on the condition that the genetic modification does not interrupt an endoge-nous gene:
  (a) targeted insertion of a contiguous DNA sequence existing in the breeder’s gene pool;
  (b) targeted substitution of an endogenous DNA sequence with a contiguous DNA sequence existing in the breeder’s gene pool;
    (**4**) targeted inversion of a sequence of any number of nucleotides;
    (**5**) any other targeted modification of any size, on the condition that the resulting DNA sequences already occur (possibly with modifications as accepted under points (1) and/or (2)) in a species from the breeders’ gene pool.

 

Source: ANNEXES to the Proposal for a REGULATION OF THE EUROPEAN PARLIAMENT AND OF THE COUNCIL (https://food.ec.europa.eu/document/download/5a994ff5-153a-4886-a3cc-794512dce27a_en?filename=gmo_biotech_ngt_proposal_2023-411_annex_en.pdf) (accessed on 25 July 2024).

The proposal was published on 5 July 2023, initiating an extensive adoption procedure. The finalized act underwent public feedback from 7 July to 8 September 2023 and was presented to the European Parliament and the Council. On 7 February 2024, the Parliament adopted the proposal, paving the way for negotiations with the Council. The entire process, from proposal negotiation to adoption of new legislation, may take between 18 months to two years.

#### 3.1.1. The Provisions of the Proposed New Legislation on NGTs in the EU

The primary objective of the proposed legislation is to establish a clear distinction between two classifications of NGT plants:

**Category 1** comprises plants that can be developed through conventional breeding methods or that occur naturally and comply with to certain equivalence criteria.
**Category 2** encompasses all other plants that are developed through NGTs failing to comply with the equivalence criteria.

The proposal requires a verification procedure to determine agreement with the equivalence criteria for categorizing NGTs into one of the two categories. For both categories, verification would be based on molecular data that confirm the absence of foreign genes. For classification to Category 1, the product should meet the equivalence criteria outlined in Box 1.

Category 1 NGT plants and products would not require risk assessment or a defined detection method. However, other Member States and the Commission retain the right to comment on the draft verification report prepared by a national competent authority. This could potentially result in a prolonged verification process, necessitating an assessment by the EFSA and the Commission, followed by a voting process among member states before the Commission ultimately reaches a final decision regarding the verification.

If a modification falls to Category 2, NGT plants and their derivatives would be accompanied by comprehensive molecular data concerning genetic alterations, also ascertaining the absence of foreign genetic material. Additional data pertaining to composition, phenotype, and toxicity/allergenicity might be necessary if there is suspicion of a plausible risk hypothesis. Category 2 plants must also be accompanied by a detection methodology compliant with GMO detection standards. In instances where providing an analytical method capable of detecting, identifying, and quantifying proves impractical, adaptations to fulfill analytical method prerequisites may be considered, provided proper justification is supplied by the notifier or applicant. The labeling of Category 2 NGT products may be supplemented with information detailing the specific trait conferred by the genetic modification.

Additionally, the proposal outlines regulatory measures designed to motivate potential notifiers or applicants for Category 2 NGT plants and products containing traits beneficial to advancing a sustainable agri-food system. These incentives aim to guide the development of Category 2 NGT plants towards traits aligned with sustainability objectives. Traits eligible for regulatory incentives encompass enhanced yield, resilience to biotic and abiotic stresses, efficient resource utilization (e.g., water and nutrients), attributes fostering sustainability throughout storage, processing, and distribution, improved quality or nutritional properties, and reduced dependence on external inputs such as pesticides and fertilizers. Notably, traits related to herbicide tolerance are excluded from regulatory incentives, including regulatory guidance or fee waivers for small- and medium-sized enterprises (SMEs). Furthermore, while Member States are allowed to prohibit cultivation of EU-approved GMOs in their territory, this does not apply to Category 2 NGT plants.

#### 3.1.2. Under the Proposed Provisions

**Category 1 NGTs** will be treated similarly to conventional varieties and will be exempt from GMO legislation requirements. There will be no need for risk assessment or labeling, and the status of these NGTs will be listed in a publicly accessible database.
**Category 2 NGTs** will be subject to regulation under current GMO legislation, necessitating risk assessment, labeling, and compliance with all other provisions of European laws governing GMO release into the environment.

Also, the Commission proposal asserts that NGT crops exempt from GMO regulations should only require labeling of their plant reproductive materials (PRM), ensuring transparency for farmers during planting, whereas the Parliament advocates for the mandatory labeling of all NGT plants upon sale to consumers.

Member States will not have the option to limit or ban the cultivation of NGT1 plants within their territories, as permitted under Directive 2001/18 for GMOs. To ensure transparency and freedom of choice for farmers, all NGT plants will be listed in a public database (Table 3).

Several amendments have already been proposed for discussions regarding the provisions of the proposal and will undoubtedly be subjected to negotiation during the final phase of deliberation in the “trilogue.” As the proposal was discussed in the Parliament, certain members advocated for the prohibition of patents on NGTs, contending that this measure would promote affordability for farmers, noting that conventionally bred plants in Europe are ineligible for patenting. Regarding patents, the Council’s stance remains undecided. Once a consensus is reached, negotiations with the Commission and Parliament will ensue. The final approval process might take from 18 to 24 months for the new legislation to materialize.

### 3.2. Considerations for Accepting Genome-Edited Crops and Their Products in the EU

Current GE crops primarily focus on improving various traits in different species, benefiting both the producers and consumers, similar to traditional GMOs [72,73]. However, the recent EU-proposed regulations differentiating SDN-1 from other GE techniques could open doors to exploring other traits or entirely new crop improvement strategies [72,74]. For example, SDN-1 technology, with its ability to introduce precise 20-base pair changes in plant genomes compared to their respective wild-type counterparts, could enable the production of valuable chemicals within plants, similar to third-generation GMOs [73]. Additionally, the combination of this targeted approach with the intrinsic ability of the DNA to act as an intelligent data storage medium [75] could potentially unlock other unforeseen crop improvement possibilities, including the development of a barcoding system for crops, similar to what has been proposed in mice [76,77].

Genome-edited foods have garnered worldwide attention, sparking debates around potential environmental and social risks. While several jurisdictions have implemented supportive regulations to facilitate their market entry [78,79], the European Union presents a unique case concerning consumer acceptance [80]. Public adoption of innovative technologies like gene editing hinges on consumer trust and understanding. A key challenge lies in the public’s tendency to conflate gene-edited foods with GMOs [81,82]. Efforts to raise public awareness about the distinct nature of gene-editing technologies are crucial to fostering consumer acceptance [83,84,85,86]. Notably, EU citizens’ awareness of gene editing has recently surpassed half the level of awareness for GMOs, but concerns surrounding this technology have also doubled [83]. This highlights the need for targeted educational initiatives to address public anxieties while promoting a balanced understanding of the potential benefits of gene editing for food security and sustainability.

The new legislation and targeted educational initiatives to address public concerns are expected to bolster trust among the public in the emerging technological capabilities, ensuring their proper application to maximize benefits while minimizing risks linked to genome editing. Judicious use of the new technology will promote research and scientific knowledge, as well as farmer and consumer wellness, in the dawn of a new era for European biotechnology that will hopefully accelerate progress and enable European countries to harvest the gains of scientific innovation for the benefit of their people.

The potential risks and regulatory challenges associated with genome-edited crops have been extensively discussed by the global scientific community, leading to proposed frameworks for their development and oversight [87,88,89,90]. Within the European Union, genome editing is primarily employed for research purposes, such as gene function validation, within certified laboratories across both public and private sectors. However, this research often falls short of translation into greenhouse or field settings. Evaluating GE crops within a plant-breeding context is crucial for translating research findings into practical applications and to avoid potential pitfalls identified in previous studies [91,92]. Notably, the United Kingdom has implemented such an approach to assess GE crops [93].

In our opinion, the implementation of successful genome-editing research with tangible outcomes that could facilitate innovation and varietal improvement within the EU could be based on the following pillars, similar to the ones proposed for international settings [88].

1. Adequate Stewardship

Current methods for genome editing in plants often involve a transient transgenic step. In this approach, foreign DNA sequences are introduced to facilitate genome modification but subsequently removed to render the final product non-transgenic. While this strategy offers non-transgenic plants, the use of a transgenic intermediate necessitates rigorous biosafety measures throughout research and development. These measures include employing molecular techniques to confirm the complete removal of transgenic elements prior to field trials, adhering to country-specific regulations for edited crops. As genome-editing technologies mature, the reliance on a transgenic intermediate stage may be circumvented. This advancement would streamline genome editing in plants, particularly for clonally propagated crops, where eliminating the intermediate transgenic elements presents a significant technical hurdle.

2. Transparency and Social Acceptance

Public trust is essential for the successful adoption of novel technologies like genome editing. A lack of transparency regarding genome-edited products can create a “social license risk” by eroding trust in developers, regulators, producers, and ultimately, the products themselves [94]. Social license, in this context, refers to the public’s willingness to accept products derived from this technology. Factors influencing social license include government policies (local regulations, global harmonization, trade, and labeling requirements) and public perception of risks and benefits. Ultimately, societal acceptance is granted by the public, both locally and globally. One key mechanism to promote transparency is an accessible, public registry. This registry would allow developers of genome-edited crops to disclose relevant information, addressing public interest in how food is produced. Importantly, these registries should be distinct from patent and regulatory risk-assessment systems. Existing examples, like The Center for Food Integrity’s registry, developed through the Coalition for Responsible Gene Editing in Agriculture [https://foodintegrity.org/programs/gene-editing-agriculture/] (accessed on 25 July 2024), demonstrate a consumer-focused approach. Such initiatives aim to address transparency concerns while incorporating the needs of the public and civil society through consumer engagement.

3. Innovation Infrastructure for Genome Editing in the EEA

The discussion of the interpretation and implementation of the variations in innovation infrastructure for genome-edited crops and other biotechnologies across European Economic Area (EEA) is still ongoing [95,96]. To expedite innovation, the EU, in collaboration with Member States, should prioritize funding and promoting research facilities suitable for genome-editing/GM research. These facilities, encompassing laboratories, greenhouses, and field sites, would serve both research and product development purposes. Crucially, the field trial infrastructure should establish a network across diverse geographical locations and climate zones. This network would facilitate the testing and evaluation of novel crop varieties under various environmental conditions, as well as the free movement inside the EU of those varieties and the products thereof. Additionally, data generated from these field trials could be directly incorporated into varietal registration processes [97].

## Figures and Tables

**Table 2:** Key steps on the way towards a revised legislation on NGT-derived plants in the EU.

Date	Authority	Type	Status
3 October 2016	French court	Preliminary ruling seeks European Court of Justice (ECJ) opinion.	OK
25 July 2018	ECJ	Released judgment.	OK
8 November 2019	European Council	Request study from Commission.	OK
4 April 2021	European Commission	Study conclusion: “Applicable legislation is not fit for purpose for some NGTs”; request for further policy reform.	OK
24 September 2021	European Commission	Policy initiative and roadmap.	OK
September 2021 to July 2022	Public consultations	Opinions and suggestions from the public.	OK
20 October 2022	EFSA	Study on risk assessment criteria for plants produced by targeted mutagenesis, cisgenesis, and intragenesis.	OK
July 2022 to July 2023	Regulatory Scrutiny Board	First mandatory impact assessment.	Negative
	Regulatory Scrutiny Board	Second mandatory impact assessment.	OK
5 July 2023	European Commission	Proposal release.	OK
7 July 2023 to 5 November 2023	Public consultations	Summary of feedback on Proposal conducted by the European Commission and presented to the European Parliament and Council.	OK
24 January 2024	EP ENVI	Vote.	OK
7 February 2024	European Parliament	Vote.	OK
The final approval process might take from 18 to 24 months	Council of EU	Agreement.	pending
	Trilogue	Negotiations between the European Parliament, the Council of EU, and the European Commission to draft the final text.	pending
	Approval in plenary and adoption	Approval.	pending

**Table 3:** Proposal provisions for the different categories of plants and products produced by all breeding methodologies.

	Conventional	NGT Category 1	NGT Category 2	GMOs
Technology	Cross-breeding; Tissue culture; Mutagenesis (chemical, radiation); Protoplast fusion (crossable species)	Genome editing (meeting equivalence criteria)	Genome editing (not meeting equivalence criteria)	Genetic engineering—Transgenics
Risk Assessment	NO	NO	adapted	YES
Labeling (proposal)	NO	PRM only	YES	YES
Labeling (Parliament)	NO	YES	YES	YES
Detection	NO	NO	adapted	YES
Member States option to refuse	NO	NO	NO	YES
Allowed in Organic farming	YES	NO	NO	NO
Info in public database	National variety register	NGT register	GMO register	GMO register
Patenting (Parliament)	NO	NO	NO	YES

## Data Availability

List of the analyzed GE studies is provided as an MS Excel file in the supporting material repository of the journal’s website. The original contributions presented in the study are included in the article/Supplementary Material, further inquiries can be directed to the corresponding author.

## Supplementary Materials

The following supporting information can be downloaded at: https://www.mdpi.com/article/10.3390/genes15081014/s1, Table S1: Comprehensive raw data on genome editing studies in plants across the world.
